# Chromosome–nuclear envelope tethering – a process that orchestrates homologue pairing during plant meiosis?

**DOI:** 10.1242/jcs.243667

**Published:** 2020-08-12

**Authors:** Adél Sepsi, Trude Schwarzacher

**Affiliations:** 1 Department of Plant Cell Biology, Centre for Agricultural Research, 2462, Martonvásár, Brunszvik u. 2, Hungary; 2 BME Budapest University of Technology and Economics, Department of Applied Biotechnology and Food Science (ABÉT), 1111, Budapest, Mu˝ egyetem rkp. 3-9., Hungary; 3 University of Leicester, Department of Genetics and Genome Biology, University Road, Leicester LE1 7RH, UK; 4 Key Laboratory of Plant Resources Conservation and Sustainable Utilization/Guangdong Provincial Key Laboratory of Applied Botany, South China Botanical Garden, Chinese Academy of Sciences, Guangzhou 510650, China

**Keywords:** Meiosis, Chromatin dynamics, Centromere associations, Synaptonemal complex, Recombination

## Abstract

During prophase I of meiosis, homologous chromosomes pair, synapse and exchange their genetic material through reciprocal homologous recombination, a phenomenon essential for faithful chromosome segregation. Partial sequence identity between non-homologous and heterologous chromosomes can also lead to recombination (ectopic recombination), a highly deleterious process that rapidly compromises genome integrity. To avoid ectopic exchange, homology recognition must be extended from the narrow position of a crossover-competent double-strand break to the entire chromosome. Here, we review advances on chromosome behaviour during meiotic prophase I in higher plants, by integrating centromere- and telomere dynamics driven by cytoskeletal motor proteins, into the processes of homologue pairing, synapsis and recombination. Centromere–centromere associations and the gathering of telomeres at the onset of meiosis at opposite nuclear poles create a spatially organised and restricted nuclear state in which homologous DNA interactions are favoured but ectopic interactions also occur. The release and dispersion of centromeres from the nuclear periphery increases the motility of chromosome arms, allowing meiosis-specific movements that disrupt ectopic interactions. Subsequent expansion of interstitial synapsis from numerous homologous interactions further corrects ectopic interactions. Movement and organisation of chromosomes, thus, evolved to facilitate the pairing process, and can be modulated by distinct stages of chromatin associations at the nuclear envelope and their collective release.

## Abstract (precis)

**Summary:** We review plant meiosis, including chromosome tethering at the nuclear periphery that, we propose, defines chromosome dynamics-facilitating DNA sequence-based pairing of homologues

## Introduction

Accurate chromosome inheritance from the diploid parental cell nucleus into haploid gametes depends on the intimate juxtaposition and recombination of paternal and maternal homologous chromosomes (see Glossary) during meiosis. As recombination through crossovers (see Glossary) involves the reciprocal exchange of genetic information between two chromosomes, conservation of genome integrity from one generation to another relies on accurate homologous partner identification.

**Glossary**

**Allopolyploid species:** species with more than one non-identical chromosome set (genome), resulting from hybridisation between two or more different species followed by genome duplication.

**Axial elements:** a meiosis-specific protein structure established along each chromosome during leptotene whereby the chromatin is organised in an array of loops with connected and co-oriented sister chromatids.

**Chiasma/chismata:** cytological manifestation of crossovers.

**Chromosome pairing:** the side-by-side alignment of homologous chromosomes prior to initiation of synapsis.

**Crossover:** site of homologous reciprocal recombination.

**Diplotene:** fourth sub-stage of meiotic prophase I, homologous chromosomes separate except at sites of chiasmata. The synaptonemal complex dissolves.

**Double-strand break (DSB):** universally the initial step in the process of meiotic recombination, involving a self-imposed DNA-damage event that is catalysed by the evolutionarily conserved protein Spo11.

**Endoreduplication:** replication of the nuclear genome without subsequent cell division, leading to a nucleus with a duplicated diploid chromosome set.

**Homoeologue/homoeologous:** partially homologous chromosomes, usually indicating some ancestral sequence homology.

**Homologue/homologous chromosomes:** have the same genetic information and order of genes, showing only allelic variation.

**Leptotene:** first sub-stage of meiotic prophase I, i.e. the period from appearance of the first elongated axial element stretches along the chromatin until their elongation, forming the continuous meiotic chromosome axes.

**Meiotic interphase:** the interphase preceding meiotic prophase I. It usually lasts longer than somatic interphases, and is characterised by chromatin re-organisation and extended protein synthesis.

**Meiotic prophase I:** the first stage of meiosis, during which pairing of homologous chromosomes and reciprocal recombination takes place. Depending on chromosome morphology, it is divided cytologically into several sub-stages (leptotene, zygotene, pachytene, diplotene, diakinesis).

**Non-disjunction of chromosomes:** unequal distribution of homologous chromosomes (meiosis I) or chromatids (meiosis II/mitosis) to daughter nuclei as a result of abnormal nuclear division.

**Nucleofilament (or presynaptic filament)**:**** filaments formed by RAD51 and DMC1 recombinases loaded into the ssDNA generated following DSBs, to execute homology search and single-end invasion (see below).

**Pachytene:** third sub-stage of meiotic prophase I, starting when synapsis is complete and synaptonemal complexes are fully formed. Chromosomes condense and bivalents (pairs of associated homologous chromosomes at meiosis I) become visible. Recombination nodules are present at sites of recombination.

**Pre-synaptic:** refers to the state of meiotic chromosomes during prophase I (typically leptotene) that shortly precedes synapsis initiation.

**Single-end invasion (SEI; also called strand invasion):** part of the DNA-repair mechanism following recombination initiation in response to DSBs. During SEI, one end of the DSB invades an intact homologous dsDNA segment to form a heteroduplex displacement loop (D-loop).

**Synapsis:** the process of intimate juxtaposition – followed by fusion – of homologous chromosomes, facilitated by the synaptonemal complex.

**Synaptonemal complex (SC):** a tripartite proteinaceous structure comprising a central element that connects the axial elements of homologous chromosomes, thus, facilitating a close chromosome juxtaposition during meiotic prophase I.

**Telomere bouquet:** characteristic gathering of telomeres within a restricted area of the nuclear periphery formed during leptotene–zygotene transition.

**Transverse filaments:** structural elements of the synaptonemal complex, which are formed by homodimers of coiled-coil proteins connected in the central region of the synaptonemal complex. Transverse filaments bring together axial elements of two homologous chromosomes.

**Triticeae:** are a tribe within the subfamily Pooideae of the Poaceae family that sometimes are referred to as small grain cereals and include many domesticated genera and species (e.g. wheat, *Triticum*; barley, *Hordeum* and rye, *Secale*, goatgrass, *Aegilops*).

**Zygotene:** second sub-stage of meiotic prophase I, characterised by initiation of synapsis between the homologous chromosomes and formation of the synaptonemal complex. Paired and unpaired chromosomes are visible.

In higher plants, hundreds of genome-wide double-strand breaks (DSBs) (see Glossary) initiate meiotic recombination within a single nucleus (Choi et al., 2013; Kurzbauer et al., 2012; Pawlowski et al., 2003). Interactions between homologous chromosomes follow recombination initiation (Bozza and Pawlowski, 2008; Hunter and Kleckner, 2001; Schwarzacher, 1997), suggesting an extremely quick and efficient activation of the homology recognition process. Moreover, in allopolyploid species (see Glossary) – such as the hexaploid wheat *Triticum aestivum* (2n=6x=42, in which x indicates the number of haploid genomes) – each homologous chromosome pair has not only one but often several highly similar, i.e. homoeologous (see Glossary), but not strictly homologous, chromosomes (e.g. AA BB DD chromosomes in wheat), with whom genetic exchange must be avoided. How homologous chromosomes recognize each other is not entirely clear yet but the paradox between the complexity of the process (Renkawitz et al., 2014) and the short time taken for pairing (see Glossary) (Barzel and Kupiec, 2008) suggests that an elaborate but reliable mechanism controlling spatial organisation of chromosomes and chromatin proximity has a significant role.

Although meiotic recombination underlies genetic diversity, the introduction of genome-wide DSBs is highly hazardous for the cell and the activation of a programmed repair mechanism is mandatory for its survival. In contrast to somatic repair, meiotic recombination utilises homologous chromosomes rather than sister chromatids to repair DSBs. Recombination is initiated by nucleolytic degradation of the 5′-termini of the broken DNAs (strand resection). The resulting single-stranded DNA (ssDNA) then attracts recombinases RAD51 and DMC1 that form nucleofilaments (see Glossary) and catalyse search and invasion into an intact homologous double-stranded DNA (dsDNA) sequence located within a partner chromosome (Pradillo et al., 2014). Current models of meiotic recombination propose that DNA sequences involved in homology searches are determined by strand resection, which typically generates 2–10 kb ssDNA, depending on the type of repair (Cannavo et al., 2019; Mimitou and Symington, 2008). During non-allelic recombination, the resulting ssDNA is longer and repair kinetics are much slower (Chung et al., 2010), indicating that homology recognition can be extended locally to a larger DNA segment. Local homology exists between non-allelic regions, especially in allopolyploids. To avoid non-allelic recombination and the resulting major rearrangements of the chromosome, it is essential to broaden homology recognition from the site of a crossover-competent DSB and apply a control mechanism based on entire chromosomes. For example, in the allotetraploid oilseed rape *Brassica napus*, homologous genomes are sorted and homologues recognise each other during the repair process that follows DSB formation (Cifuentes et al., 2010; Howell et al., 2008). Crossovers are formed between chromosomal regions with the closest sequence homology; however, when lacking a homologous partner, recombination is promoted between homoeologues (Grandont et al., 2014). This can be explained by the presence of a nuclear recognition system, in which recombination intermediates with the highest level of homology are favoured over the weakest recombination intermediates.

Here, we summarise the current understanding of meiotic chromatin dynamics in plants, by connecting recent cytological and molecular information, to explore and advance our understanding of the role of meiotic chromosome behaviour in homologous pairing. Several recent reviews have highlighted the possible function of early, non-homologous centromere associations in meiosis (da Ines and White, 2015) and the different pairing strategies observed in other eukaryotes (Loidl, 2016; Zickler and Kleckner, 2015), as well as the movement of meiotic chromosomes directed by nuclear envelope-associated proteins (Zeng et al., 2018). The present Review adds to these studies by examining telomere and centromere-led chromatin dynamics in relation to formation of the synaptonemal complex (SC) (see Glossary) and meiotic recombination. Building on our own research in wheat and barley (Schwarzacher, 1997; Sepsi et al., 2017, 2018) as well as other studies of plants, we argue that specific steps of synapsis (see Glossary) progression are modulated by the mechanical constraints imposed by the tethering and associations of telomeres and centromeres to the nuclear envelope. Our discussion complements the interpretation of recent molecular data, e.g. on chromatin positioning at the nuclear periphery as reported in *Arabidopsis thaliana* reproductive tissues (Bi et al., 2017), and is relevant in understanding the function of chromatin dynamics in plants in general but also of those in non-plant eukaryotes.

## Nuclear envelope bridge complexes – a mechanism ensuring chromatin tethering and movement during meiotic prophase I

**Fig. 1.**
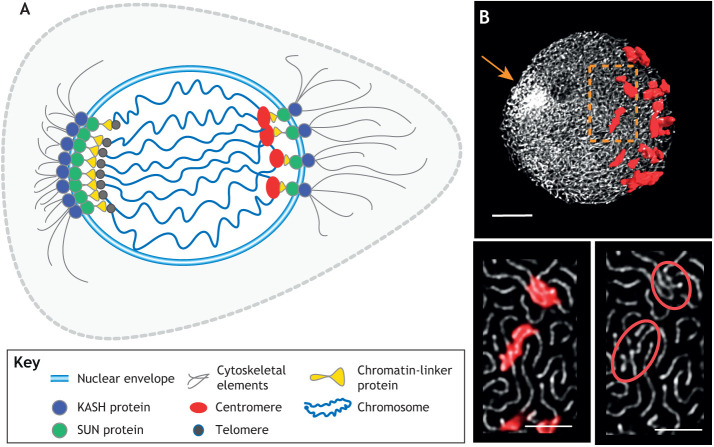
**Mechanism ensuring centromere and telomere tethering to the nuclear envelope.** (A) Schematic representation of a cell nucleus during early meiosis (leptotene), with chromosomes attached to the nuclear periphery at centromeres and telomeres by adaptor proteins and the linker of the nucleoskeleton and cytoskeleton (LINC)-complex. Notice that, although proteins ensuring meiotic telomere–nuclear envelope tethering are well reported, only limited knowledge is available about the factors that ensure meiotic centromere–nuclear envelope tethering and, therefore, the present image represents our hypothesis on the basis of studies involving *S. cerevisiae* interphase cells (Hou et al., 2012, 2013; Braun and Barrales, 2016). (B) Top: 3D rendering of *z*-stack images showing centromere arrangements at the nuclear periphery of a wheat meiotic nucleus. Scale bar: 5 µm. Centromeres are associated with each other at early meiosis (leptotene) at the nuclear periphery, as detected by immuno-labelling for the centromeric histone H3 (CENH3; red) and for the chromosomal axis-related protein ASY1 (shown as white threads). The telomere bouquet is visible as a strongly stained area opposite to the centromeric pole (arrow). Bottom: magnified views of the boxed area of the top image. Single *z*-stack shows the organisation of chromosome axes at high resolution with groups of centromeres are located at the nuclear periphery. Scale bars: 2.5 µm.

To achieve chromatin movements while chromosomes pair in the meiotic nucleus surrounded by the double membrane system of the nuclear envelope cytoskeletal mechanical forces need to be transmitted from the cytoplasm to the nucleoplasm, bypassing the barrier formed by the nuclear envelope (Evans et al., 2011). Indeed, chromosome motion during prophase I is achieved by cytoplasmic motor proteins (kinesin and myosin in plants; kinesin, myosin and dynein in animals), which convert the chemical energy from ATP hydrolysis into mechanical force (Nebenführ and Dixit, 2018). Motor proteins can actively generate movement along the cytoskeleton, such as the actin cables that surround the nucleus [e.g. *Saccharomyces cerevisiae*, Koszul et al., 2008; *Z. mays*, Sheehan and Pawlowski, 2009) and microtubules (e.g. *Drosophila melanogaster*, Christophorou et al., 2015; *mus musculus*, Lee et al., 2015). The transfer of mechanical energy between the cytoplasm and nucleoplasm is implemented by nuclear envelope bridge proteins referred to as linker of the nucleoskeleton and cytoskeleton (LINC) complex (Chikashige et al., 2007; Rothballer and Kutay, 2013), which spans the nuclear envelope, extends into the nucleoplasm and connects to the chromatin (Fig. 1A). Here, we present a short overview of the LINC complex elements (for more details, we refer to recent reviews by Evans et al., 2014; Pradillo et al., 2019; Zeng et al., 2018; Zhou and Meier, 2013).

### Composition of the LINC complex in plants

The core components of the LINC complex are the Sad1 and UNC-84 homology (SUN) and Klarsicht/ANC-1/Syne-1 homology (KASH) domain-containing proteins. SUN-domain proteins, which are highly conserved within eukaryotes, are integrated into the inner nuclear membrane through their transmembrane segment (Hagan and Yanagida, 1995), whereas their N-terminus is exposed to the nucleoplasm (Conrad et al., 2007; Malone et al., 1999) and interacts with the chromatin through chromatin-binding proteins. KASH-domain proteins are incorporated into the outer nuclear membrane by their transmembrane helix; their divergent N-terminus is exposed to the cytoplasm where it connects to cytoskeletal elements, i.e. actin, intermediate filaments and microtubules (Apel et al., 2000; Starr and Han, 2002). SUN-domain proteins, thus, link the chromatin to the cytoskeleton through their evolutionarily highly conserved C-terminal SUN-domain that extends into the perinuclear space and directly binds its outer nuclear membrane partners, the KASH-domain proteins (Sosa et al., 2012; Zhou et al., 2012a). In plants, SUN-domain proteins were characterised in detail in *A. thaliana* (*At*SUN1–*At*SUN5) (Graumann et al., 2010, 2014). In *Zea mays*, a search for SUN-domain proteins (Murphy et al., 2010) revealed two classes. (i) Two SUN-domain proteins comprising a C-terminal SUN domain (*Zm*SUN1, *Zm*SUN2), which both are structurally similar to those in animals and fungi, and (ii) three, so-called, plant-prevalent SUN-domain transmembrane proteins that comprise a mid-SUN3 domain (*Zm*SUN3, *Zm*SUN4, *Zm*SUN5). These two classes of SUN-domain proteins are also present in other flowering plants, and phylogenetic analysis suggests ancient divergence of the two subclasses (Murphy et al., 2010). In *A. thaliana*, C-terminal SUN-domain proteins (*At*SUN1 and *At*SUN2) were shown to interact with KASH-domain proteins in the perinuclear space (Graumann et al., 2014; Zhou et al., 2014). In contrast, mid-SUN-domain proteins interact with other mid-SUN-domain proteins as well as with C-terminal SUN-domain proteins, and it has been proposed that they are involved in chromatin anchorage to the inner nuclear membrane (Graumann et al., 2014). Unlike SUN-domain proteins, KASH-domain proteins show a remarkable lack of conservation. Genes encoding KASH-domain proteins have been identified in a wide range of animals [*Mus musculus* (Horn et al., 2013); *Caenorhabditis elegans* (McGee et al., 2006; Starr and Han, 2003); *Drosophila* (Technau and Roth, 2008)] but also in plant genomes [*A. thaliana* and *Z. mays* (Meier, 2016; Zhou and Meier, 2013)]. Specifically, tryptophan-proline-proline (WPP)-domain interacting proteins (Xu et al., 2007), WPP-interacting tail-anchored (WIT) proteins (Zhou et al., 2012b) and SUN-domain-interacting nuclear envelope (SINE) proteins (Zhou et al., 2014) were identified as plant-specific outer nuclear membrane proteins. Recently, a new grass-specific KASH protein family has also been reported in *Z. mays* (Gumber et al., 2019), including the *Z. mays* LINC complex proteins KASH grass-specific 1 (*Zm*MLKG1) and *Z. mays* LINC KASH *At*WIP-like 1 and 2 (*Zm*MLKP1 and *Zm*MLKP2) proteins, which were shown to interact with the inner nuclear membrane protein *Zm*SUN2 at the nuclear periphery.

### Functions of cytoskeleton and LINC complex during meiotic prophase I

The severe meiotic aberrations observed in the absence of SUN-domain proteins uncovered their fundamental role in meiotic progression in both *A. thaliana* and *Z. mays* (Murphy et al., 2014; Varas et al., 2015a). The complex between SUN- and KASH-domain proteins (Fig. 1A) is involved in meiotic telomere–nuclear envelope tethering in *A. thaliana* (Varas et al., 2015a) and meiotic telomere dynamics are correlated with *Zm*SUN2 in *Z. mays* (Murphy et al., 2014). For instance, *Zm*SUN2 colocalises with the nuclear envelope at leptotene (see Glossary) and forms a full belt-like structure around the nucleus. Following a dynamic nuclear reorganisation at zygotene (see Glossary), it becomes restricted to the nuclear area – ‘half-belt’ – that includes the telomere bouquet (see Glossary) (Murphy et al., 2014). Importantly, although spatial polarisation of specific classes of SUN-domain protein in the nucleus at meiosis has not been reported in other plant species, the enrichement of actin and microtubule arrays at the outer nuclear membrane during prophase I (*Lycopersicon esculentum* and *Ornithogalum virens*: Hogan, 1987; *Solanum melongena*: Traas et al., 1989; *Gasteria verrucosa*: Van Lammeren et al., 1985; *Gagea lutea*: Bohdanowicz et al., 2005) and the association of microtubules with chromatin (Sheldon and Dickinson, 1986) suggest that the cytoskeleton carries meiosis-specific functions. Recent time-lapse imaging in *A. thaliana* (Prusicki et al., 2019) revealed progressive polarisation of the microtubule array in late leptotene, when the bouquet is formed (Hurel et al., 2018). Microtubule polarisation led to the formation of an arc structure (also known as half-moon structure) on one side of the nucleus, whereas the nucleus itself moved towards the corner of the cell (Prusicki et al., 2019). Asymmetrical positioning of bouquet-stage nuclei and polarisation of the microtubule array was also observed in rye, where the telomere bouquet oriented towards the corner of the cell, away from the majority of the microtubules (Cowan et al., 2002). The majority of the microtubules, thus, surrounded the periphery of the opposite nuclear hemisphere, i.e. one half of the nuclear volume, a territory occupied by polarised centromere associations in wheat (Fig. 1B), rye and other members of the *Triticeae* tribe – i.e. small grain cereals, such as barley, oat, and triticale – at meiotic prophase (see Glossary) (Mikhailova et al., 2001; Phillips et al., 2012; Sepsi et al., 2018).

### Proteins ensuring centromere- and telomere attachments to the LINC complex

In many organisms, initiation of meiotic prophase I (see Glossary) is marked by centromere–centromere associations close to the nuclear periphery and by the assembly of the telomere bouquet at the opposite nuclear pole. Centromere–centromere associations were shown to rely on centromere activity in both plants (Zhang et al., 2013) and *Drosophila* (Unhavaithaya and Orr-Weaver, 2013); however, the crucial components that link centromeres to the nuclear envelope are yet to be discovered in plants. In fission yeast interphase cells, centromere clustering and tethering is executed by the nuclear adaptor protein Csi1, which interacts both with centromeres and Sad1, i.e. the inner nuclear envelope SUN-domain protein (Hou et al., 2012). Meiosis-specific adaptor proteins between telomeres and the LINC complex are well known in yeast, i.e. Taz1, Rap1 and Bqt1 to Bqt4 in *S. pombe* (Chikashige and Hiraoka, 2001; Chikashige et al., 2006., 2009) and Ndj1 in *S. cerevisiae* (Conrad et al., 2008) as well as mammals, i.e. TERB1/2 and MAJIN (Morimoto et al., 2012; Shibuya et al., 2015) – but they need yet to be identified in plants.

Recent progress in the identification and characterisation of SUN- and KASH-domain proteins in plants uncovered the basis of chromosome positioning at the nuclear envelope, a prerequisite for its movement during meiotic recombination and synapsis. Identification of the crucial elements that ensure chromatin tethering to the LINC complex in plants will provide the possibility to generate viable mutant lines, and help us gain a better understanding of the role centromere and/or telomere tethering have during meiosis.

## Presynaptic chromatin dynamics in prophase I – progressive nuclear polarisation through centromere clustering and the telomere bouquet

Before discussing chromatin dynamics during synapsis and recombination in plants, we provide a short overview of the key molecular and cytological events during various stages of meiotic prophase I, which are defined by chromosome morphology (see definition of meiotic prophase I in Glossary; for a more-detailed discussion of early meiosis, please see recent reviews by Lambing et al., 2017; Mercier et al., 2015; Osman et al., 2011; Pradillo et al., 2014; Zickler and Kleckner, 2015).

### Meiotic recombination initiation – an overview

**Fig. 2.**
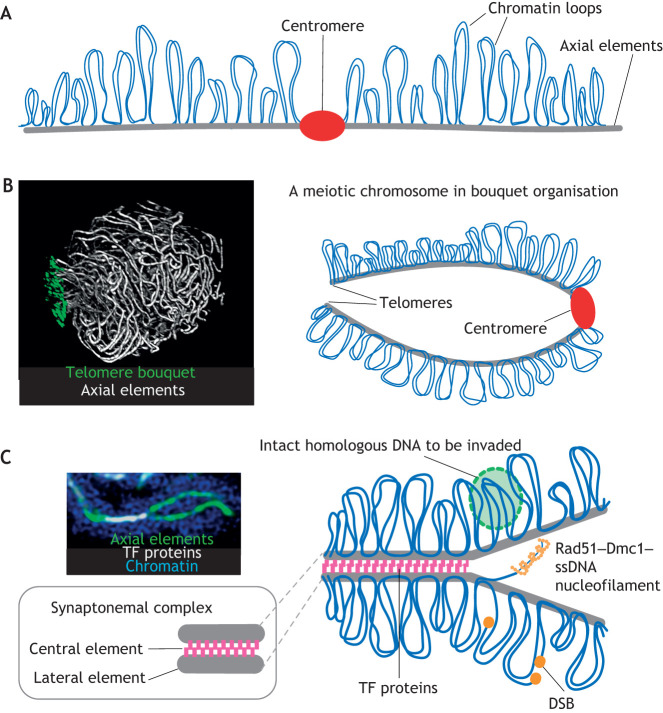
**Structure and organisation of meiotic chromosomes.** (A) Schematic representation of a meiotic chromosome with the chromatin organised as an array of loops (blue) along a protein axis (axial elements, grey). The centromere is shown in red. (B) Chromosome arrangement inside the nucleus during early meiosis (leptotene). Left: Microscopic image, showing a 3D-rendered leptotene nucleus of tetraploid barley, with fully elongated axial elements (grey) and telomeres attached to a restricted area – the telomere bouquet (green) – of the nuclear periphery. Notice the apparent parallel alignments of the subtelomeres, i.e. the area of the chromosome arm in the vicinity of the telomeres. Right: Schematic drawing, showing the arrangement of a single meiotic chromosome (as shown in A) when enclosed into the nucleus during late leptotene. The chromosome forms a large loop, as the telomeres gather and assemble the chromosome bouquet. The centromere is located at the opposite pole of the nucleus and involved in associations with other centromeres during leptotene (not shown). (C) Organisation of the synaptonemal complex between two homologous chromosome segments. Left: Microscopic image, showing two parallel axial elements (green) along unsynapsed chromatin (blue) surrounding them. The axial elements are partially connected by the transverse filament (TF) proteins of the synaptonemal complex (white). Right: Schematic image, showing an array of chromatin loops (blue) along axial elements (grey) of the synaptonemal complex that is progressively connected by TF proteins (purple) that form the central element of the synaptonemal complex. Axial elements are referred to as lateral elements once they are connected by TFs (see magnified view, bottom left). Meiotic double-strand breaks (DSBs) are formed within chromatin loops, and the, subsequently, generated single-strand DNA (ssDNA) attracts the strand-exchange proteins (DMC1 and RAD51) to form nucleofilaments (orange coil) (see Glossary) and initiate SEI into an intact homologous DNA segment (green circle) on the homologous partner chromosome.

In plants, early events of meiotic prophase I take place in the presence of a meiosis specific chromosome axis (Crismani et al., 2013; Ferdous et al., 2012; Li et al., 2011; Sanchez-Moran et al., 2007). The meiotic axis formation involves the installation of synaptonemal complex axial elements (see Glossary) along the chromatin, which is organised as an array of loops (Fig. 2) with sister chromatids connected and co-oriented (Zickler and Kleckner, 1999). Axis proteins play an important role in the choice of recombination partners by favouring inter-homologue over inter-sister recombination (Ferdous et al., 2012). During formation of the meiotic chromosome axis in higher plants, recombination is initiated by DSBs (Higgins et al., 2012), catalysed by SPO11, a homologue of the A subunit of the archaebacterial topoisomerase VI complex (Keeney et al., 1997; Szostak et al., 1983). Of the three SPO11 proteins encoded by *A.* *thaliana At*SPO11-1 and *At*SPO11-2 are involved in meiotic DNA cleavage (Stacey et al., 2006), whereas *At*SPO11-3 has a role in somatic endoreduplication (see Glossary) (Hartung et al., 2007). DSBs are followed by 5′-3′ DNA end resection, and the obtained 3′ single-strand overhangs attract disrupted meiotic cDNA 1 (DMC1) and radiation sensitive 51 (RAD51) strand-exchange proteins (RecA in prokaryotes), which form nucleoprotein filaments and initiate single-end invasions (SEIs) (see Glossary) into an intact homologous duplex DNA (Fig. 2C and Hunter and Kleckner, 2001). In *A. thaliana*, *At*DMC1 (Klimyuk and Jones, 1997; Couteau et al., 1999), together with axis proteins, regulates inter-homologous recombination bias, whereas *At*RAD51 ensures the fidelity of homology recognition (Pradillo et al., 2012).

Initiation and progression of meiotic recombination in *A. thaliana* requires the presence of axis proteins ASY3 (Ferdous et al., 2012) and ASY1 (Sanchez-Moran et al., 2007), respectively. ASY4, another protein related to the axis, is dispensable for SEI but required for the maturation of recombination intermediates into crossovers (Chambon et al., 2018).

### Centromere polarisation in early leptotene

**Fig. 3.**
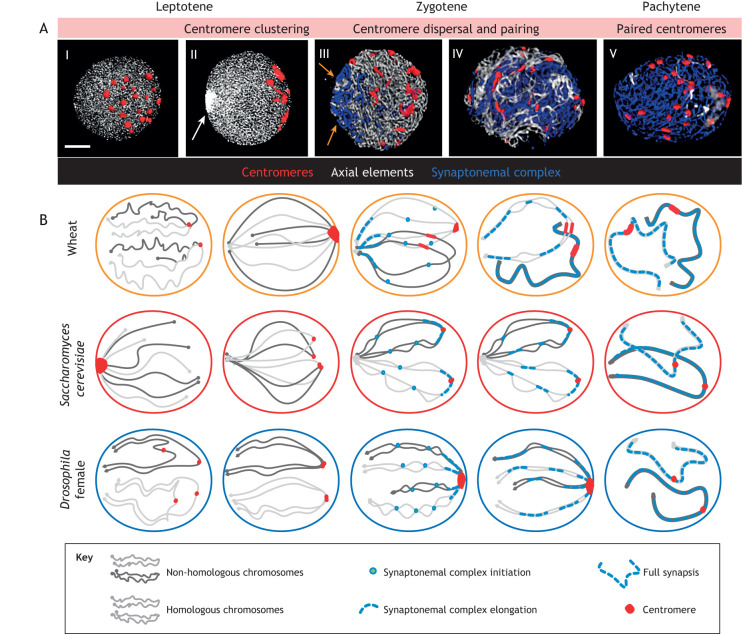
**Strategies of homologous chromosome pairing in selected higher plants, *S. cerevisiae* and insects. (**A) Reconstructed immunofluorescence micrographs that show the dynamic behaviour of the centromeres in hexaploid wheat (2n=42) during synaptonemal complex formation at meiotic prophase I. (I) Early leptotene. Centromeres (red) form numerous small groups (red dots) within the nuclear periphery; axial elements of the synaptonemal complex are present as short stretches (light grey). (II) Late leptotene. Centromeres show the characteristic spatial polarisation at one pole of the nucleus (large red dots) and are associated within few large groups (notice that wheat has 42 individual centromeres), the telomere bouquet being at the opposite nuclear pole (arrow). Image reproduced from Sepsi et al. (2018) under the CC-BY licence. (III) Early zygotene. The synaptonemal complex starts to elongate (indicated by arrows) from the region adjacent to the telomeres (subtelomeres); centromeres (red) become separated from each other and form the nuclear periphery. As synapsis progresses, axial elements (grey) are replaced with central elements (blue). Interstitial regions of the chromosome arms do not show synapsis. (IV) Mid to late zygotene. Formation of interstitial regions synapse and release of the spatial polarisation (formed earlier through centromere associations and the initial steps of synapsis) of the nucleus. The end of polarisation is apparent from the quasi-random localisation of the synapsed chromosome segments and the scattered centromeres. (V) Pachytene. Completed (full) synapsis; axial element proteins have been replaced by transverse filament proteins (blue threads). Scale bar: 5 µm. (B) Top row: Schematic representation of chromosome pairing in wheat showing two example chromosomes. Middle row: centromeres in *S. cerevisiae* cluster at the spindle pole body at interphase (centromere clustering) from where they disperse upon meiotic initiation. The spindle pole body is then occupied by telomeres that form the bouquet, and non-homologous centromeres become associated (non-homologous centromere coupling). Non-homologous centromere coupling switches to homologous centromere pairing, tightly followed by centromeric synaptonemal complex initiation (centromeric synapsis). This is followed by interstitial synaptonemal complexes elongating from multiple sites (interstitial synapsis). Bottom row Centromere pairing in female *Drosophila* is achieved gradually during mitotic cycles that precede meiotic initiation (chromosome alignment and centromere pairing). The centromere cluster is formed in early zygotene and centromeric synaptonemal complex formation is initiated. The interstitial synaptonemal complex elongates later, initiating from multiple nucleation sites (interstitial synapsis).

In several organisms, meiotic interphase (see Glossary) chromosomes are arranged in a Rabl orientation, i.e. a non-random arrangement of chromosomes, where centromeres and telomeres are positioned peripherally, in opposite nuclear hemispheres (Schwarzacher, 1997; Tiang et al., 2012). Additionally, in some species, centromeres associate in small groups (Fig. 3A) in the vicinity of the nuclear envelope in the interphase preceding meiotic prophase and during leptotene (e.g. *S. cerevisiae*: Jin et al., 1998; *Tetrahymena thermophyla*: Loidl et al., 2012; *H. sapiens*, *M. musculus*: Scherthan et al., 1996; onion: Church and Moens, 1976; *Triticum aestivum*: Bennett and Smith, 1979; Naranjo and Corredor, 2004; Sepsi et al., 2018; *Hordeum vulgare*: Phillips et al., 2012; rye: Mikhailova et al., 2001; *Z. mays*: Zhang et al., 2013). Within the plant kingdom, i.e. in *Brachypodium* (Wen et al., 2012) and wheat (Martinez-Perez et al., 2003), small centromere groups further associate, which culminates in extreme clustering (peripherally localised, large, polarised centromere groups, Fig. 3B), a phenomenon suggested to facilitate the homologous pairing of chromosomes. For instance, Martinez-Perez et al. (2003) hypothesised that, when clustering in hexaploid wheat, centromeres become connected as homologous pairs and contribute to the homology recognition process. This model has, however, been challenged by several studies that demonstrated that polarised, early centromere associations are mainly non-homologous in monocotyledons (hereafter referred to a monocots; Corredor et al., 2007; Dvorak and Lukaszewski, 2000; Phillips et al., 2012; Prieto et al., 2004; Sepsi et al., 2017), although homologous interactions may also take place (Schwarzacher, 1997). Interpretation of 3D centromere dynamics together with immunolocalisation of the synaptonemal complex proteins in hexaploid wheat indicated that early centromere behaviour exerts a mechanistic role in homologous pairing, and defines the organisation of bulk chromatin during specific steps of synapsis and meiotic recombination (Sepsi et al., 2017). Collective centromere tethering at the nuclear pole in wheat and barley occurs while axial elements elongate along the length of chromosomes (early leptotene; Sepsi et al., 2018) and, thus, coincides with DSB formation (Higgins et al., 2012). During this period, centromere clusters anchored to a specific area of the nuclear envelope generate a semi-polarised nuclear environment, in which chromosomes are arranged as large loops that are attached to the nuclear periphery (Fig. 3). In *Z. mays*, peripheral centromere associations are also formed (during meiotic interphase to leptotene) and rely on specific elements of the sister-chromatid cohesion complex cohesin and on the structural maintenance of chromosome 6 homologue protein (SMC6). The latter has been proposed to be a component of the synaptonemal complex in *Z. mays* (Zhang et al., 2013), showing that presynaptic centromere behaviour is regulated by synaptonemal complex proteins in monocots. Colchicine − which specifically disrupts the spindle microtubules (Bennett and Smith, 1979) − has no effect on presynapstic centromere associations, indicating that their maintenance is independent of microtubules (Corredor and Naranjo, 2007).

Similarly, in *A.* *thaliana*, centromeres are peripherally located during early meiosis; they are widely dispersed and appear unassociated during leptotene but, during zygotene, non-homologous centromere associations arise that, as meiosis progresses, transition to homologous centromere pairing (Ronceret et al., 2009). Non-homologous centromere associations are manifested in *A. thaliana* *spo11-1*, *rad51*, *dmc1* and *rad51 dmc1* mutants (da Ines et al., 2012), revealing that such associations depend neither on SPO11-induced DSBs, nor the SEI process. Recombination initiation in plants, thus, occurs within a partially restricted chromatin state that is imposed by the tethering of centromere–centromere associations to the nuclear envelope (Triticeae) or the formation of non-homologous centromere–centromere associations within the nuclear space (*A. thaliana*).

### The function of the telomere bouquet during homologous chromosome pairing

Following centromere polarisation and during the elongation of the axial elements at leptotene (Sepsi et al., 2017), the first DSBs are processed into SEIs and directed motion of the telomeres progressively forms the telomere bouquet (Higgins et al., 2012). As a result, chromosomes become coherently arranged and stretched between the two poles (Fig. 3) (Cowan et al., 2002; Scherthan, 2001), a state that most probably limits robust chromatin movements and defines chromosome trajectories. In addition to enabling spatial chromatin polarisation, formation of the telomere bouquet is involved in the initiation of SUN- and KASH-domain protein-dependent telomere-led meiotic chromosome movements within fission yeast (Chikashige et al., 2007), budding yeast (Trelles-Sticken et al., 2005), mice (Morimoto et al., 2012) and plants (Murphy et al., 2014; Varas et al., 2015b). The inner nuclear membrane protein SUN2 colocalises with the telomere bouquet in *Z. mays* and has been proposed to mediate the telomere arrangement at the nuclear periphery as well as telomere-led chromosome movements (Murphy et al., 2014). Telomere-led rapid chromatin motion in plants has only been visualised using live cell imaging in pollen mother cells of *Z. mays* (Sheehan and Pawlowski, 2009) and involves dynamic rotations of the bulk chromatin, i.e. movement of short, individual chromosome segments and deformation of the nuclear envelope. The complex patterns of prophase motion suggest that it is generated by different types of cytoplasmic motility force rather than being implemented by a single predominant force-generating mechanism. For instance, the telomere bouquet itself, as one specific feature of chromosome movement, forms and disperses independently of cytoplasmic microtubules and has been suggested to rely on a yet-unknown, probably tubulin-related, protein (Cowan and Cande, 2002). Chromosome movements in plants have been proposed as one mechanism to facilitate homologous loci recognition during zygotene (Golubovskaya et al., 2002; Sheehan and Pawlowski, 2009) and remove connections between entangled chromosomes as shown in *A. thaliana* (Martinez-Garcia et al., 2018). Although chromosome pairing (see Glossary) in budding yeast relies on rapid prophase movements rather than the telomere bouquet itself (Lee et al., 2012), in higher plants the bouquet is considered a significant element of pairing, as demonstrated in meiotic mutants defective of telomere bouquet formation, showing impaired synapsis and recombination (Golubovskaya et al., 2002). In the *pairing homologous1* mutant (*Ph1^−^*) wheat line, non-homologous (homoeologous) pairing correlates with a delay in telomere bouquet formation (Richards et al., 2012), showing that the bouquet has a role regarding fidelity of pairing.

Polarised chromosome organisation created by centromere and telomere associations with the nuclear envelope, thus, implies that chromosomes move as tethered loops inside the meiotic nucleus. In most organisms, chromosome arm ratios (defined by centromere positions) vary within heterologous and homoeologous chromosomes (Heslop-Harrison and Bennett, 1983; International Wheat Genome Sequencing Consortium (IWGSC), 2014; Lukaszewski et al., 1982; Maccaferri et al., 2019). Consequently, chromatin movements constrained by centromere and telomere tethering result in different mechanical properties of non-homologues, and result in decreased affinity of chromosome arms compared with that of true homologues. The tethered chromosome conformation reinforces different velocities of non-homologues during prophase movements and, thus, allows initial testing of chromosomal similarity on a simple mechanical basis during SEI, while promoting proximity for homologues.

## Chromatin dynamics during synapsis

Synapsis, the intimate juxtaposition and progressive connection of axial elements by transverse filaments (see Glossary), yields a highly ordered structure, i.e. the synaptonemal complex (Page and Hawley, 2004) (Fig. 2C). Meiotic recombination maturation in plants occurs within the stable construct of the synaptonemal complex and crossover maturation happens in coordination with synaptonemal complex morphogenesis (Cahoon and Hawley, 2016; Sanchez-Moran et al., 2007). The fidelity of chromosome interactions is dramatically affected by the absence of the synaptonemal complex in *A. thaliana*, leading to multivalent chromosome associations and non-homologous chiasma (see Glossary) formations (Higgins et al., 2005). Depletion of the main structural synaptonemal complex component (ZYP1) in barley results in significantly reduced chiasma formation, chromosome non-disjunction (see Glossary) and loss of fertility (Barakate et al., 2014). Here, we review general strategies of synapsis initiation and meiotic recombination in plants and explore the underlying chromatin dynamics as a possible mechanism that evolved to support and validate homology recognition between chromosomes.

### Emergence of the synaptonemal complex follows the polarised pattern of recombination initiation

In higher plants, a minority of DSBs result in reciprocal exchange between proportions of one chromatid of each of the two homologous chromosomes, i.e. crossovers. The majority of recombination initiation sites, however, result in non-reciprocal exchange and is resolved with non-crossovers (Grelon, 2016). Crossover and non-crossover formation events rely on single-end invasions and serve as synapsis initiation sites (Zhang et al., 2014); they are, however, spatiotemporally distinct and highly controlled. In the tribe of *Triticeae* (see Glossary), reciprocal exchange occurs within the sub-telomeres (Darrier et al., 2017), whereas in *A. thaliana* the highest rate of crossovers appears to be in the pericentromeric regions (Rowan et al., 2019). The decision whether to follow a crossover or non-crossover pathway for crossover processing is decided at an early stage of meiosis (Bishop and Zickler, 2004) and both structures play vital roles at meiosis. Crossovers by securing genetic diversity and accurate chromosome segregation and non-crossovers by ensuring that synapsis is initiated at many points along the chromosome axes where homology has been determined by DNA-dependent homology recognition.

In the fungus *Sordaria macrospora* (Storlazzi et al., 2008), the transition from chromosome alignment to synaptonemal complex nucleation is mediated by robust inter-axis bridges that include axis components, recombination proteins and the evolutionarily conserved Zip2–Zip4 complex. These recombination complexes are located between the aligned axes and form bridges that, ultimately, serve as synaptonemal complex nucleation sites (Dubois et al., 2019; Zhang et al., 2014). Synapsis requires chromosome axis remodelling in *A. thaliana*, that includes the depletion of the axis-associated protein ASY1, mediated by the pachytene checkpoint homolog 2 (PCH2) protein (Chambon et al., 2018; Lambing et al., 2015) an ATPase from the family of ATPases associated with diverse cellular activities (AAA+). ASY1 depletion from the chromatin is, thus, fundamental for synaptonemal complex polymerisation and correct crossovers maturation.

The mature synaptonemal complex is composed of a central element that is made up of transverse filaments and additional proteins, and positioned between two synapsed axial elements (referred to as lateral elements) (Hesse et al., 2019; Higgins et al., 2014). In many plants, immunostaining of the synaptonemal complex central-element protein ZYP1 uncovered that the first synaptonemal complex stretches appear adjacent to telomeres (Fig. 3; see also Colas et al., 2017), which in some species corresponds to the first sites of recombination initiation (Blokhina et al., 2019; Higgins et al., 2014). As sub-telomeric regions pair, interstitial regions remain initially dissociated (Higgins et al., 2012; Zhang et al., 2013) but, later, form a synapse with synaptonemal complexes, which elongate from multiple sites (Sepsi et al., 2017) and may include recombination intermediates that later resolve as non-crossovers. During synapsis initiation and progression in hexaploid wheat nuclei expand and unsynapsed axes actually become more distant – interaxis distances are between 300 and 500 nm compared with less than 300 nm at leptotene – (Sepsi et al., 2018). Assuming that synapsis initiation requires axis proximity, the relaxed axis distances at zygotene suggest that the majority of interstitial SC initiations are formed earlier (Sepsi et al., 2018). Synaptonemal complex tracts progressively coalesce and form full synaptonemal complexes at pachytene followed by homologue desynapsis at diplotene (see Glossary), leading to the physical manifestation of mature crossovers as chiasmata. (see Glossary) (Fig. 3; Higgins et al., 2012; Schwarzacher, 1997). Distinct genetic control of distal and interstitial synapses in plants has been demonstrated through meiotic mutations that specifically affect the synapsis of internal telomeric and sub-telomeric regions in *Z. mays* (Golubovskaya et al., 2002).

### Centromere release from the nuclear periphery coincides with main events of synapsis initiations

As mentioned above, pre-synaptic (see Glossary) chromatin arrangements involves tethering polarised chromosomes at the nuclear periphery through centromere clustering and the telomere bouquet. In a recent study, we have shown that, in bread wheat, sub-telomeric synapsis initiation coincides with the dispersion of centromere clusters into the nuclear space (Sepsi et al., 2017). During this, partially released, chromatin state – where telomeres are still gathered into the bouquet – the interstitial synaptonemal complex elongates. Resolution of the centromere clusters is gradual and mirrored by the gradual juxtaposition of chromosome arms with synapsis finally progressing to the centromeres. The precise coordination of nuclear envelope-related centromere dynamics with synapsis progression suggests a role for centromere release during interstitial synapsis and recombination. The attachment of centromeres to the nuclear envelope, as seen during leptotene, reduces the large-scale chromatin motility and favours chromosome–chromosome interactions that are necessary for DSB-dependent homology recognition (Sepsi et al., 2017).

**Fig. 4.**
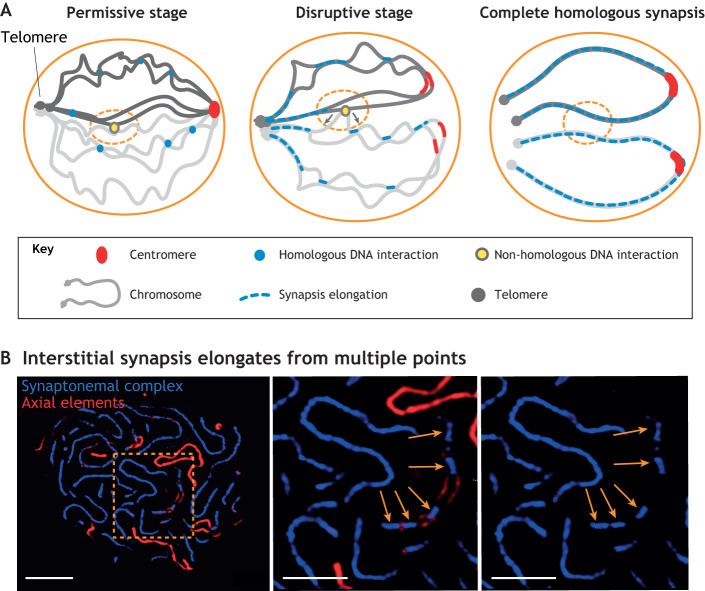
**Elimination of** **ectopic chromosome interactions in higher plants that** **comprise** **large chromosomes.** (A) Schematic diagram showing the resolution of non-homologous DNA interactions during meiotic prophase I. Permissive stage: Double-strand break (DSB)-dependent chromosome interactions initiate during leptotene; however, these interaction are error prone and both homologous and non-homologous (ectopic) contacts are formed. Ectopic interactions are due to partial similarity between sequences located on non-homologous chromosome arms. Synapsis in plants (and many other organisms) is first initiated by elongation from subtelomeres, providing a stable attachment of the chromosome arms while centromeres become dispersed from the nuclear periphery. Disruptive stage: Elimination of several ectopic interactions is achieved by the higher motility of chromosome arms upon centromere release, whereas stable attachment at the telomeres ensures coherent movement of the homologues. Subsequently, this allows interstitial synaptonemal complexes to elongate from multiple homologous points within one chromosome arm and the small number of remaining ectopic interactions will be disrupted by the synaptonemal complex itself, assuming that longer synaptonemal complexes are formed between homologous partners. Complete homologous synapsis: Ectopic pairing is resolved and synaptonemal complexes are formed along the entire length of homologous chromosomes. (B) Left: Nucleus of the wheat ‘Maris Huntsman’ at late zygotene. The boxed area shows a short (5 µm) segmented, parallel region of two chromosome axes (ASY1 staining, red) undergoing synapsis (ASY1 staining interspaced with staining against ZYP1, blue). Several synaptonemal complex initiation sites are visible within a short (∼5 µm) chromosomal stretch (∼5 µm). Scale bar: 5 µm. Middle, right: Magnification of the boxed area within the image on the left. Each blue segment (indicated by arrows) represents at least one synapsis initiation point that, during elongation, connects the two parallel axial elements (red) (middle image). Right: Single-channel blue image. Scale bars: 2.5 µm.

What, then, is the importance of dissociating centromeres from the nuclear envelope? After the elongation of subtelomeric synapsis and during the formation of interstitial synapsis the release of the centromeres allows a more-robust movement that can interrupt eventual non-homologous interactions and promotes the elongation of the synaptonemal complex from stable, truly homologous DNA connections. Release of centromere tethering has been shown to considerably increase chromatin motility in centromere proximal regions within budding yeast interphase cells (Verdaasdonk et al., 2013). If centromere release leads to an increased motility at zygotene while telomere-led chromosome movements persist, it might, indeed, effectively separate weakly associated, non-homologous interactions (Conrad et al., 2008; Penkner et al., 2009; Sato et al., 2009). Partially homologous regions anneal with less affinity than perfect homologues and are, thus, more prone to be disrupted by robust movements. A model on the basis of polymer physics demonstrated that the range of chromatin motion can be altered efficiently by attaching or detaching the chromatin from a tether (Verdaasdonk et al., 2013). We propose that cytoskeletal forces transmitted to the telomeres as well as telomere-led chromosome movement, together with centromere tethering, exert an affinity discrimination function at meiosis (Fig. 4). There, similarities between chromosomes can initially be tested on a simple physical basis, e.g. chromosome lengths and arm ratios become prominent in the tethered state, which contributes to the fidelity of the DNA-dependent homology search.

Although centromere dynamics related to nuclear envelope attachment have only been addressed in a few plant species, the progressive transition from early non-homologous to homologous centromere pairing appears to be conserved among plants. As interstitial synapsis progresses in *A. thaliana*, non-homologous centromere associations switch to homologous pairing (da Ines et al., 2012; Pradillo et al., 2014; Ronceret et al., 2009). Centromere pairing was found to rely on DMC1, whereas interstitial synapsis extension demands the presence of RAD51 (da Ines et al., 2012). Thus, homologous centromere pairing requires recombination initiation and a mature synaptonemal complex but has different requirements regarding recombination proteins compared with pairing of chromosome arms. The different requirements for chromosome arm and centromere pairing have also been demonstrated in budding yeast, where non-homologous centromere association occurs and centromeric synapsis, subsequently, is initiated in a manner that depends on the member of the synapsis initiation complex Zip2 (Chuong and Dawson, 2010). Synapsis initiation sites within chromosome arms are formed later in *S. cerevisiae* (Fig. 3) and depend on Zip3 – another component of the synapsis initiation complex – demonstrating that centromeric and interstitial synapses are temporally separated and have distinct genetic regulators (MacQueen and Roeder, 2009). In female *Drosophila*, where the telomere bouquet is not formed, synapsis starts at clustered centromeres, followed by further ‘waves’ of synaptonemal complex initiation at multiple points within the euchromatic chromosome arms (Fig. 3; see also Liu et al., 2002; Takeo et al., 2011; Tanneti et al., 2011). Centromeric- and interstitial synapses are controlled separately by two functionally unique meiosis-specific cohesin complexes, whereby the centromeric synaptonemal complex depends on the ORD–SOLO–SUNN protein complex and interstitial synapsis has a strong requirement for the cohesion components crossover suppressor on 2 of Manheim [C(2)M], stromalin (SA) and nipped-B (Gyuricza et al., 2016).

The distinct genetic control of synapsis at centromeres, telomeres and chromosome arms, together with the fact that arm synapsis emerges from numerous foci, suggests an evolutionarily selective advantage for the mechanism of multiple-step pairing (Fig. 4). Separation of sub-telomeric and interstitial pairing in plants provides multiple levels of control over homology assessment. Synapsis at the subtelomeres provides structural stability for chromosome ends and ensures that telomere-led movements can effectively act on the interstitial regions to separate ectopic interactions, where homologous chromosome arms are connected through multiple events of SEI and embedded synaptonemal complex nucleation. Accordingly, extensive synapsis would only form between homologues. The striking centromere and telomere associations, and the rapid chromosome movement that precedes initiation of the interstitial synaptonemal complex – as discussed here – are crucial in this process; they create a mechanical context that can rapidly and effectively modulate timing of the spatially separated pairing in sub-telomeric regions and at chromosome arms.

### Elimination of ectopic interactions – a model

In this Review, we propose a model where chromatin dynamics, in tight coordination with synaptonemal complex formation, function as an effective system to eliminate ectopic interactions during plant meiosis (Fig. 4). Initial DNA–DNA interactions at leptotene are formed during the permissive stage, where physical properties of chromosomes – such as chromosome length, chromosome arm ratios, and their tethering at the nuclear envelope – imply synchronous movements between homologues and different relative velocities or pace of movement between heterologous chromosomes. Although this favours homologous contacts, ectopic interactions can also be made in this context owing to the tight packaging of the nucleus, which imposes a multitude of chromosome contacts. Initial pairing is strongly position-dependent in plants and many other eukaryotes, in which it occurs mostly at telomeres. It is determined by telomere-led active motion (Marshall and Fung, 2016), leading to sub-telomeric synaptonemal complex initiation during which interstitial formation of synaptonemal complexes is attenuated – although the underlying molecular mechanism needs yet to be identified in plants. Elongation of the interstitial synaptonemal complex occurs later, starting at several loci, and occurs while centromeres are released from the nuclear envelope. We hypothesise that the resulting enhanced chromosome arm motility and larger nuclear volume at zygotene compared to that during leptotene allows movement that effectively eliminates ectopic DNA interactions during interstitial synaptonemal complex elongation.

The remaining recombination intermediates formed between homologous chromosomes will allow synaptonemal complex elongation and, thus, provide a mechanical platform to further eliminate any remaining ectopic interactions – i.e. the disruptive stage. Maturation of synaptonemal complexes relies on multiple homology recognition sites along the chromosomes and is highly accurate in determining homology of entire chromosomes. During correct meiosis in plants, early events of recombination promote correct synapsis of homologues and formation of the synaptonemal complex. Once the latter has reached its final size (i.e. when the homologous synapsis is complete at Pachytene, the homologous synapsis stage), it promotes the maturation of the recombination intermediates into crossovers (Henderson and Keeney, 2005; Zhang et al., 2014; Barakate et al., 2014; Colas et al., 2016). Full synapsis, thus, converts local homology to homology at a nucleus-wide level, endorsing homology of whole chromosomes and, ultimately, homology of entire genomes.

## Conclusions

The identification of key players of recombination, synapsis and chromatin remodelling in plants during the last two decades has greatly advanced our understanding of the plant meiotic process. Here, we have discussed how to integrate crucial features of chromatin dynamism into our knowledge of homologous recombination and chromosome pairing. On the basis of our own research on the *Triticeae*, as well as other recent studies of plants, we argue that, to understand the function of chromatin dynamics, centromere and telomere movement should be assessed at the same time as synapsis progression and recombination. The wide accessibility of high-resolution microscopy and optical sectioning, together with cytological techniques for the 3D analysis of meiotic nuclei, allows to decipher specific nuclear features and their relation to the nuclear envelope in remarkable detail. Further investigations of centromere dynamics during key events of meiosis, using a wide variety of plant species will help to unveil conservation and divergence between different taxonomical groups. Molecular studies aimed at identifying key components of the LINC complex and of adaptor proteins that ensure chromatin–nuclear envelope tethering, will generate possibilities to construct mutant plant lines. Such studies will also be useful to test the function of tethering in processes, such as regulation of crossover distribution in higher plants and regulation of crossover inhibition within the pericentromeric regions.
